# The prognostic value of homocysteine levels in hemorrhagic stroke patients: a systematic review and meta-analysis

**DOI:** 10.3389/fneur.2025.1576453

**Published:** 2025-04-28

**Authors:** Yi Zong, Qiaohui Gu

**Affiliations:** ^1^Department of Emergency, Huzhou Third Municipal Hospital, The Affiliated Hospital of Huzhou University, Huzhou, Zhejiang, China; ^2^Department of Neurology, Huzhou Traditional Chinese Medicine Hospital Affiliated to Zhejiang Chinese Medical University, Huzhou, Zhejiang, China

**Keywords:** hemorrhagic stroke, homocysteine, meta-analysis, prognosis, systematic review

## Abstract

**Background:**

Hemorrhagic stroke is associated with significant morbidity and mortality, prompting the search for modifiable risk factors and prognostic indicators. Elevated homocysteine levels have been implicated in vascular diseases, but their link to hemorrhagic stroke outcomes remains unclear. This systematic review and meta-analysis aimed to evaluate the association between homocysteine levels and outcomes in hemorrhagic stroke patients.

**Methods:**

The MEDLINE, EMBASE, and Cochrane Central databases were searched for studies comparing the outcomes of hemorrhagic stroke in patients with high versus normal homocysteine levels. Data were pooled using random-effects models to calculate odds ratios (ORs) for mortality and poor functional outcomes and standardized mean differences (SMDs) for homocysteine levels, with respective 95% confidence intervals (CIs).

**Results:**

The review included 10 studies. Pooled analysis showed no significant association between high homocysteine levels and increased risk of mortality (OR: 1.123, 95% CI: 0.589 to 2.143), poor functional outcomes (OR: 1.203, 95% CI: 0.962 to 1.504), or unfavorable neurological outcomes (OR: 1.001, 95% CI: 0.618 to 1.620). Substantial heterogeneity was observed among studies.

**Conclusion:**

High homocysteine levels were not significantly associated with mortality, functional outcomes, or unfavorable neurological outcomes in hemorrhagic stroke patients. The clinical utility of homocysteine as a prognostic marker in this population remains uncertain and warrants further research.

**Systematic review registration:**

https://www.crd.york.ac.uk/PROSPERO/, CRD42024527207.

## Introduction

Hemorrhagic stroke is associated with particularly high mortality and morbidity rates (1). Unlike ischemic stroke, which results from blockages in blood flow to the brain, hemorrhagic stroke is caused by ruptured blood vessels, leading to blood accumulation that can cause brain cell damage (2). The management and prognosis of hemorrhagic stroke remain challenging, highlighting the need for identifying reliable prognostic markers that could guide therapeutic strategies and improve patient outcomes (3).

Recently, the potential prognostic value of homocysteine in the context of hemorrhagic stroke has become a focus of research (4, 5). Elevated homocysteine levels, a condition known as hyperhomocysteinemia, have been implicated in various pathological processes, including endothelial dysfunction, oxidative stress, and inflammatory responses, all of which can exacerbate vascular damage and contribute to the risk of hemorrhagic stroke (5, 6). Moreover, hyperhomocysteinemia is associated with other vascular risk factors, such as hypertension, diabetes, and dyslipidemia, which are prevalent among stroke patients (7–9).

The potential link between homocysteine levels and hemorrhagic stroke outcomes comes from the role of homocysteine in vascular biology. The research suggests that homocysteine may contribute to the weakening of blood vessel walls, leading to an increased propensity for rupture and bleeding within the brain (10). Elevated homocysteine levels, therefore, might not only increase the risk of stroke but also influence recovery, functional outcomes, and the likelihood of recurrent bleeding (11–13). Nevertheless, the prognostic value of homocysteine in patients with hemorrhagic stroke is still unclear. While some studies report a direct correlation between high homocysteine levels and poor post-stroke outcomes, such as higher mortality rates and severity of the condition, others fail to establish a significant association (11–13). This discrepancy could be attributed to differences in study design, population characteristics, timing of homocysteine measurement, and the heterogeneity in defining outcomes. This review aims to critically evaluate and synthesize existing research on the prognostic value of homocysteine in patients who have suffered a hemorrhagic stroke.

## Methods

### Inclusion criteria

#### Participants

This review included studies with adult patients (aged 18 years and older) diagnosed with hemorrhagic stroke.

#### Exposure

The primary exposure of interest is elevated homocysteine levels in patients diagnosed with hemorrhagic stroke.

#### Comparator

The comparator group consists of hemorrhagic stroke patients with normal or lower levels of homocysteine. Studies without clear categorization based on homocysteine levels or lacking comparative homocysteine levels data between exposure and comparator groups were excluded.

#### Outcome

Primary outcomes are mortality rates, functional outcomes as measured by scales such as the Modified Rankin Scale (mRS—0–2 indicates good outcome, 3 and above indicates poor outcome), and favorable or unfavorable neurological outcomes (severe intracranial hemorrhage or low Glasgow coma scale score or low Glasgow outcome score).

#### Study design

The review included randomized controlled trials, cohort studies, case–control studies, and observational studies. Case reports, editorials, reviews, and animal studies were excluded.

### Exclusion criteria

Studies focusing exclusively on pediatric populations (patients younger than 18 years of age); Studies that did not report homocysteine levels or failed to compare elevated versus normal/lower levels of homocysteine among hemorrhagic stroke patients; Studies that did not report on the primary outcomes of interest, such as mortality rates, functional outcomes, or neurological outcomes; Studies without a clear diagnosis of hemorrhagic stroke or those including mixed stroke populations without separate analysis for hemorrhagic stroke patients; Case series, case reports, editorials, commentaries, conference abstracts, and reviews without original data.

#### Information sources and search strategy

The search for pertinent studies was carried out across various electronic databases, registers, and additional resources: MEDLINE (through PubMed), EMBASE, Cochrane Library, Web of Science, and Scopus. Additionally, manual searches of reference list from key studies and review articles were performed. Targeted searches of websites of relevant professional organizations and societies in the fields of neurology and stroke, such as the American Heart Association (AHA) and the World Stroke Organization (WSO), were also conducted. The comprehensive search strategy aimed to encompass literature from the inception of the databases until February 2024 without any language filter.

#### Study selection process

Two independent reviewers thoroughly evaluated the literature to ensure a comprehensive and unbiased selection. Titles, abstracts, and key terms of studies, identified by the literature search were screened. This preliminary screening was followed by an in-depth review of the full-text articles to verify their suitability based on predetermined inclusion criteria. In instances of disagreement between the reviewers, discussions were held to reach a consensus. The review was conducted in accordance with PRISMA guidelines (14).

#### Data collection

Data were extracted by the principal investigator and included bibliographic details such as the date of extraction, titles, and authors’ names, as well as specific study characteristics including design, participant demographics, and context. Particular emphasis was placed on extracting detailed information regarding the study cohorts, outcome measures at the start and end of the studies, the criteria for participant inclusion and exclusion, intervention specifics, control groups, and duration of follow-up. Both primary and secondary outcome data, the timing of these assessments, and other relevant factors for assessing study quality were meticulously recorded. To ensure the reliability of this process, a second reviewer independently verified the extracted information against the source documents.

#### Risk of bias

Newcastle-Ottawa Scale (NOS) was used to assess the quality of observational studies (15). This scale evaluates observational research through three main dimensions: the selection criteria of study participants, the comparability between different groups, and the accuracy in determining the exposure for case–control studies or the outcome for cohort studies. Each study can receive maximal score of 9, with higher score indicating superior methodological quality.

### Statistical analysis

The meta-analysis used STATA software version 14.2 (StataCorp, College Station, TX, United States). For binary outcomes, the number of events in both the exposed (higher homocysteine levels) and non-exposed (normal or lower homocysteine levels) groups from each stud were recorded, and pooled odds ratios (ORs) were calculated along with 95% confidence intervals (CIs) to estimate the combined effect size. For continuous outcomes, mean values and standard deviations for each group were extracted and the pooled standardized mean differences (SMDs), accompanied by 95% CIs, were computed to assess the effect magnitude across studies (16).

Heterogeneity was assessed by the I^2^ statistic and the chi-square test (15). Additionally, sensitivity analyses were conducted where possible to determine the robustness of our findings. Due to the limited number of included studies, publication bias was not assessed.

## Results

### PRISMA search results

**Figure 1:**
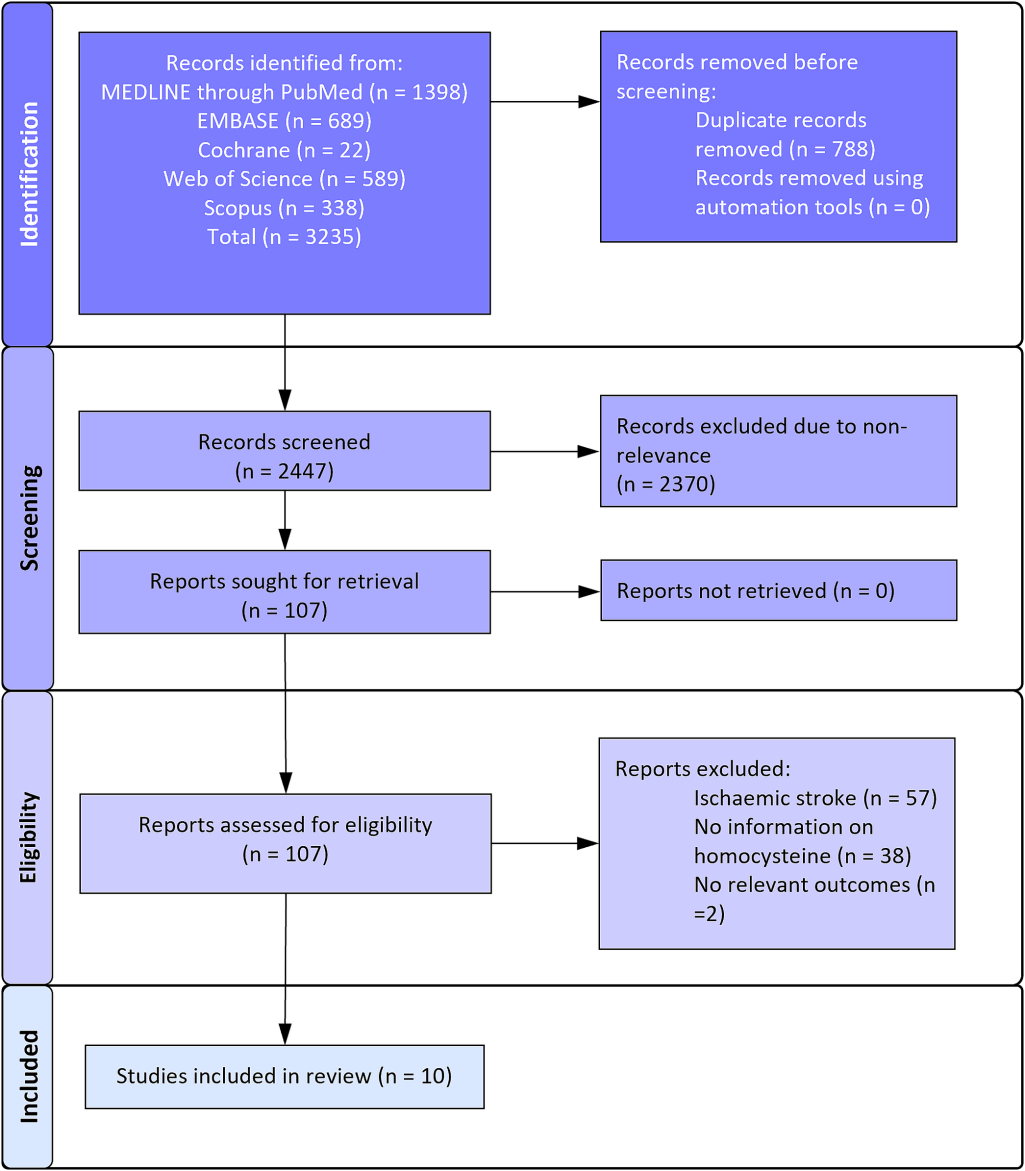
PRISMA flowchart.

Overall, 3,235 records were retrieved across all the databases. Of them, 788 duplicates were removed and 2,477 records underwent primary title and abstract screening. A total of 2,370 records were excluded in this stage and 107 full texts were retrieved to check for the final eligibility. Ultimately, 10 studies were eligible for the analysis (Figure 1) (11–13, 17–23).

### Characteristics of the included studies

The review encompassed 10 studies, predominantly conducted in China (eight studies) and India (two studies), reflecting significant Asian demographic representation. The studies varied widely in sample size, ranging from 69 to 85,705 participants, demonstrating diverse research scopes and population scales. The mean age across studies predominantly fell within the fifth to sixth decade of life, aligning with the typical age range for hemorrhagic stroke occurrence. Based on the risk of bias assessments, five studies were categorized as having a low risk of bias, while other five studies had moderate to high risk, indicating potential limitations in their design or reporting (Table 1).

**Table 1:** Included studies (*n* = 10).

First author and year of publication	Study design	Study country	Description of participant details	Sample size	Follow up duration	Outcomes reported	Homocysteine cut-off	Mean age in years	Gender distribution	Risk of bias
Zhou et al. 2015 (23)	Retrospective cohort study	China	Patients admitted within 24 h from the onset of acute spontaneous ICH.	69	6 months	Poor neurologic outcome	14.62 μmol/L	61 ± 11	45/24	Low
Wang et al. 2020 (5)	Prospective cohort study	China	ICH was diagnosis by the WHO standard and confirmed by the hospital’s computerized tomography (CT) scan with age ≥ 18 years old and arriving at hospital within 72 h after onset which is first ever acute-onset ICH.	551	1 month, 3 months, and 1 year separately	Poor functional outcome (death or disability)	15 μmol/L	HHcy = 58 (48–67) Normal Hcy = 57 (50–65)	386/165	Moderate
Dhandapani et al. 2015 (12)	Prospective cohort study	India	Patients with spontaneous subarachnoid hemorrhage admitted within 5 days of ictus	90	3 months	Neurological outcome Mortality	15 μmol/L	49	45/45	Low
Suo et al. 2018 (20)	Retrospective cohort study	China	Patients aged 18 years or older with ICH	263	72 h	Poor neurologic outcome (haemotoma expansion)	Mean value among participants (exact value NR)	53.4 ± 14.0	201/62	High
Li et al. 2021 (13)	Prospective	China	Patients with hemorrhagic stroke	84	6 months	Glasgow Outcome Scale (GOS) (Mild defect but good recovery and a normal life, Mild disability and independence, severe disability and life need others to take care, Survival in a vegetative state, death)	Analyzed as continuous variable (instead of cut-off)	I = 63.4 ± 6.3 C = 61.6 ± 5.4	48/36	Low
Dai et al. 2021 (11)	Cross-sectional study	China	Patients with hemorrhagic stroke and having the complete data on baseline GCS and tHcy measurements.	1,516	No follow up	Poor neurologic outcome (GCS level)	10 μmol/L	61.1 ± 12.4	976/540	High
Liu et al. 2016 (17)	Retrospective case–control study	China	Aneurysmal subarachnoid hemorrhage (aSAH) group: aSAH confirmed by DSA at admission or surgery and serum parameters obtained within the first 24 h after admission. Non-aSAH group: patients with intracerebral hemorrhage and patients complicated with renal dysfunction, atherosclerosis, stroke, myocardial infarction, or diabetes mellitus.	2,175	Admission to discharge	Poor neurologic outcome reported as Glasgow Outcome Scale (GOS)	14.62 μmol/L	I = 57.24 ± 13.0 C = 55.77 ± 9.7	78/172	Moderate
Wang et al. 2022 (21)	Secondary data analysis from a hospital registry	China	Patients were 18 years or older and primary diagnosis of ICH confirmed by brain CT or MRI.	85,705		Severe ICH, hospital mortality, and a poor functional outcome at discharge.	15 μmol/L	62.3 ± 13.1	34,705/21,088	Moderate
Sagar et al. 2021 (19)	Prospective cohort study	India	ICH onset (within 72 h), aged between 18 to 85 years and resident within North India	323	3 months	Mortality (mRS: 6) and poor clinical outcome (mRS: 3–6) at 3 months. Good Outcome (mRS = 0–2)	14.61 μmol/L	54.9 ± 12.8	162/88	Low
Peng et al. 2022 (18)	Prospective cohort study	China	Patients with primary intracerebral hemorrhage admitted within 24 h after symptom onset.	724	3 months	Poor functional outcomes Mortality	24.9 μmol/L	61.43 (11.5)	404/175	Low

### Mortality

**Figure 2:**
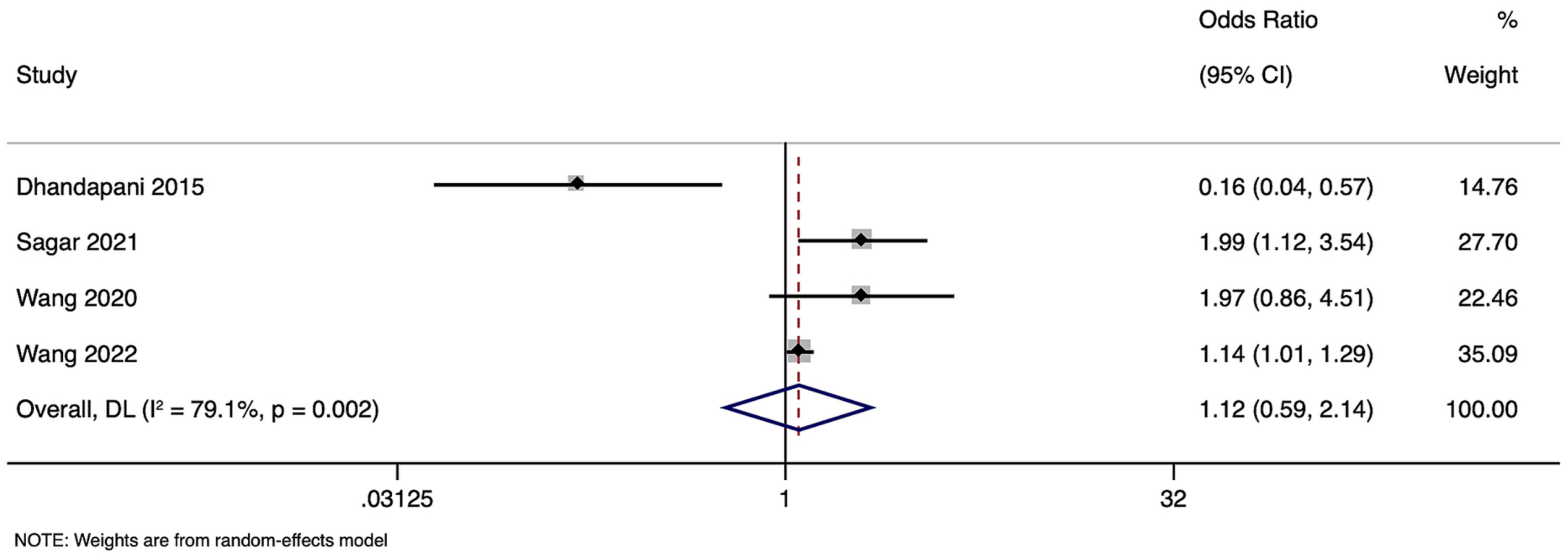
Forest plot showing the difference in mortality between high and normal levels of homocysteine.

Four studies with 56,647 participants were included in the meta-analysis of mortality. Pooled OR for the risk of mortality in patients with high homocysteine levels compared to those with normal levels was 1.123 (95% CI: 0.589 to 2.143), indicating comparable risk. However, substantial heterogeneity was observed among the studies (I^2^ = 79.1%, *p* = 0.002), suggesting considerable variability in study outcomes.

(Figure 2). Sensitivity analysis showed no difference in the estimates (Supplementary Figure 1).

### Functional outcome

**Figure 3:**
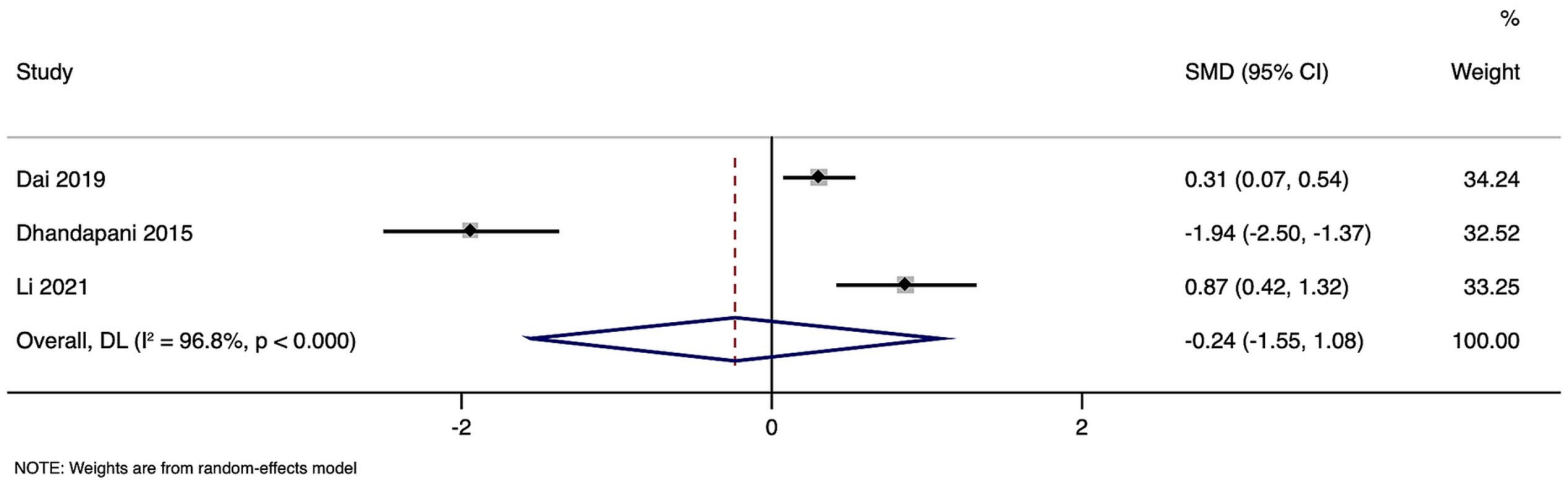
Forest plot showing the difference in functional outcome between high and normal levels of homocysteine.

Meta-analysis of functional outcomes included five studies with 57,242 participants. Pooled OR for poor functional outcomes in individuals with high homocysteine levels compared to those with normal levels was 1.203 (95%CI: 0.962 to 1.504) (Figure 3). This result suggests a non-significant increase in the risk of poor outcomes associated with high homocysteine levels. The analysis revealed moderate heterogeneity among the studies (I^2^ = 48.1%, *p* = 0.103), indicating some variation in the study findings. Sensitivity analysis showed no difference in the estimates (Supplementary Figure 2).

### Unfavorable neurological outcome

**Figure 4:**
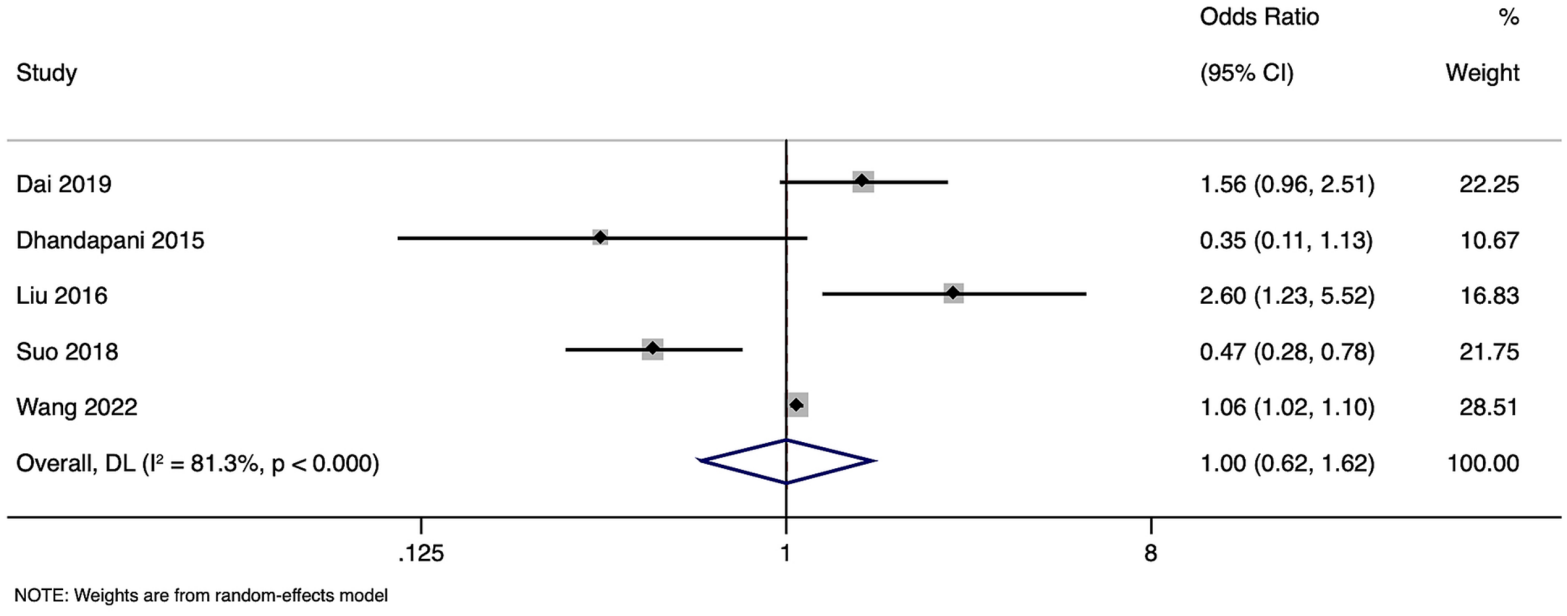
Forest plot showing the difference in poor neurological outcome between high and normal levels of homocysteine.

Meta-analysis of unfavorable outcomes combined data from five studies with a total of 57,770 participants. The pooled OR of the association between high homocysteine levels and unfavorable neurological outcomes was 1.001 (95% CI: 0.618 to 1.620) (Figure 4), indicating a lack of significant difference in patients with high or normal homocysteine levels. Notably, the heterogeneity was significant (I^2^ = 81.3%, *p* < 0.001), suggesting considerable variation in outcomes among the included studies. Sensitivity analysis showed no difference in the estimates (Supplementary Figure 3).

**Figure 5:**
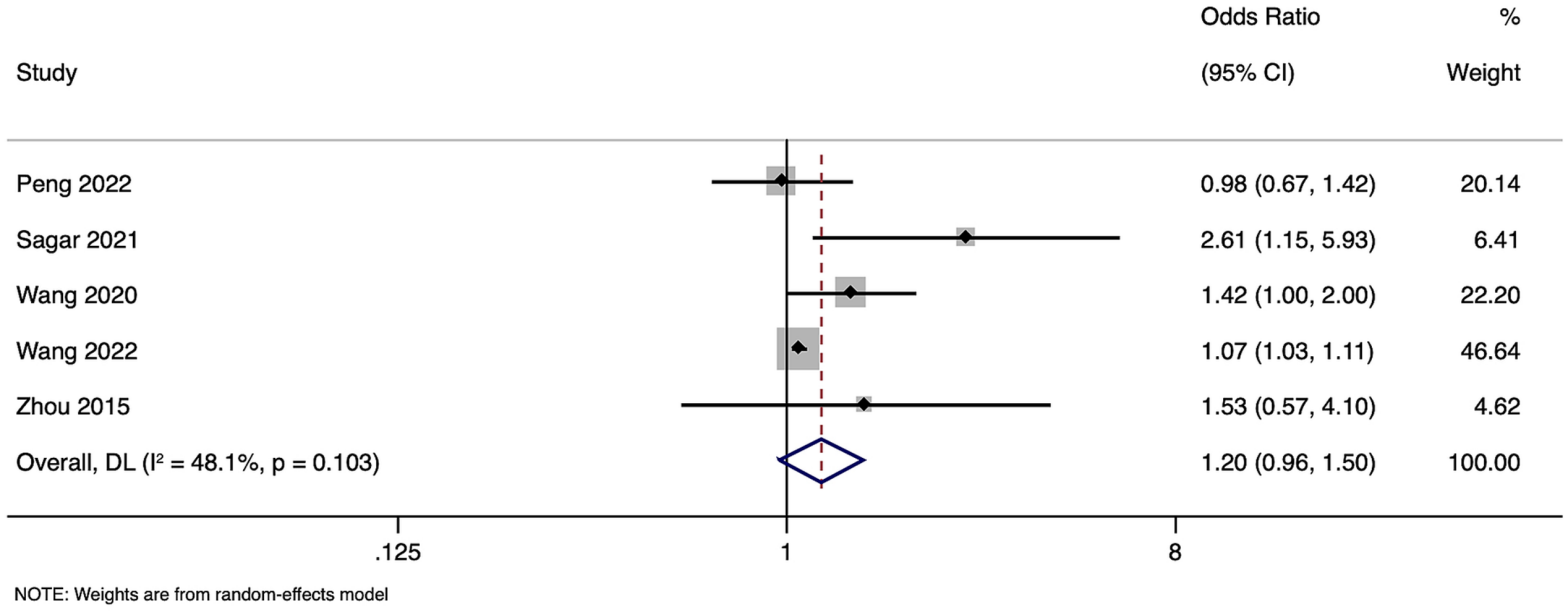
Forest plot showing the difference in homocysteine levels between good and poor neurological outcome.

In this meta-analysis, three studies with a total of 1,485 participants were analyzed to assess the SMD in homocysteine levels between groups with unfavorable and favorable outcomes. Pooled SMD was −0.236, with a 95% CI ranging from −1.551 to 1.078 (Figure 5). This result suggests comparable homocysteine levels in the two groups, as indicated by the *p*-value of 0.724. However, the analysis showed a high level of heterogeneity among the studies (I^2^ = 96.8%, *p* < 0.001), indicating substantial variability in the effect sizes of the individual studies.

## Discussion

This systematic review and meta-analysis evaluated the relationship between high homocysteine levels and various outcomes in patients following a hemorrhagic stroke, incorporating data from multiple studies with a total of 56,647 participants for mortality risk, 57,242 for functional outcomes, and 57,770 for unfavorable neurological outcomes. Our findings reveal several critical insights. A pooled OR of 1.123 for mortality risk between individuals with high versus normal homocysteine levels did not show a significant difference. This suggests that while homocysteine levels may be a marker of interest in the context of hemorrhagic stroke, they might not directly influence mortality outcomes.

The analysis indicated a non-significant increase in the risk of poor functional outcomes in patients with elevated homocysteine levels (OR = 1.203). Although not statistically significant, this increasing trend points toward a potential link between homocysteine levels and the quality of recovery post-stroke, which should be addressed by further research.

Our findings revealed no significant association between high homocysteine levels and the risk of unfavorable neurological outcomes (OR = 1.001), indicating that homocysteine levels might not be a reliable prognostic marker for neurological recovery post-hemorrhagic stroke. Pooled SMD of −0.236 between groups of patients with unfavorable and favorable outcomes further highlighted similar homocysteine levels. Substantial heterogeneity was observed across studies for mortality and unfavorable neurological outcomes. Such heterogeneity may be due to the variability in methodologies and populations among the included studies. In contrast, studies reporting data on the functional outcomes showed moderate heterogeneity.

The relationship between homocysteine levels and hemorrhagic stroke outcomes has been a subject of debate in the neurological and cardiovascular fields. While some studies reported a significant association between elevated homocysteine levels and increased risks of mortality and morbidity post-stroke, others found no such relationship (11–13, 17–24), which is similar to our observations. Our study found no significant correlation between elevated homocysteine levels and increased mortality, functional deficits, or unfavorable neurological outcomes in patients after hemorrhagic stroke. The discrepancy between our findings and those of studies showing a significant relationship (24–28) may be attributed to the differences in patient demographics, stroke severity, timing of homocysteine measurement, or even the definitions of high homocysteine levels across studies. Additionally, hemorrhagic stroke differs fundamentally from ischemic stroke and other cardiovascular diseases, where homocysteine has been more consistently implicated. Therefore, the mechanisms through which homocysteine affects vascular integrity and function may not directly influence the outcomes of hemorrhagic events, which are more related to immediate physical damage and subsequent pressure effects (23).

The prognostic value of homocysteine may critically depend on the timing of the homocysteine measurement post-stroke. Acute phase reactions, changes in renal function, and various interventions may all alter homocysteine levels, influencing their relationship with outcomes (29).

Individual differences in the homocysteine metabolism, possibly influenced by genetic factors, nutrition, and other lifestyle factors, could also affect the apparent impact of homocysteine levels on stroke outcomes across a diverse patient population (25–29).

Additionally, the biochemical pathways linking homocysteine to vascular health might exhibit a threshold effect, beyond which further increases in homocysteine levels do not exacerbate risk or where only extreme elevations significantly impact hemorrhagic stroke outcomes (13). Moreover, the role of homocysteine in neuroinflammation and oxidative stress, which are considered key factors in secondary brain injury following hemorrhagic stroke, is still poorly understood. Exploring these mechanisms could uncover new insights into the interaction between elevated homocysteine levels and brain recovery processes. It is also conceivable that the impact of homocysteine on hemorrhagic stroke outcomes is moderated by factors such as the extent of hematoma expansion, the presence of intraventricular hemorrhage, or patient-specific responses to hyperhomocysteinemia, such as variations in the methylenetetrahydrofolate reductase gene, which could influence individual susceptibility to the detrimental effects of high homocysteine levels (21–23).

### Strengths

Our review covers a large sample size, enhancing the statistical power and generalizability of the findings. The inclusion of studies with diverse populations and settings provides a comprehensive view of the literature. Rigorous methodological standards, such as the use of the Newcastle-Ottawa Scale for quality assessment, add to the reliability of our review.

### Limitations

High heterogeneity among included studies suggests variability in study designs, patient populations, and outcome measurements, which could impact the overall findings. The exclusion of non-English language studies may introduce language bias. The observational nature of included studies limits the ability to infer causality between homocysteine levels and stroke outcomes.

### Study implications

Our findings suggest that homocysteine levels alone should not currently be used for prognostic purposes in hemorrhagic stroke patients. Clinicians should continue to focus on established clinical indicators and interventions while considering homocysteine as part of a broader assessment of vascular risk factors.

Future studies should standardize the timing and methods for measuring homocysteine levels post-stroke. Future research can also explore potential interactions between homocysteine and other biological or clinical factors that may influence stroke outcomes. Further studies should focus on the effects of interventions targeting homocysteine levels on post-stroke recovery and long-term outcomes. Future research should include larger, more diverse populations to confirm our findings and perform various subgroup analyses.

## Conclusion

This review indicates that there is no significant association between elevated homocysteine levels and mortality, functional outcomes, or unfavorable neurological outcomes in hemorrhagic stroke patients. Despite substantial heterogeneity across studies, our findings contribute to a better understanding of the role of homocysteine in the prognosis of hemorrhagic stroke. Given the complexity of stroke recovery and the multifactorial nature of outcomes, homocysteine levels should not be considered in isolation. Further research is needed to clarify the potential role of homocysteine and to develop targeted therapeutic strategies for improving outcomes in hemorrhagic stroke patients.

## Data Availability

This data can be found at: the MEDLINE, EMBASE, and Cochrane Central databases were searched for studies comparing the outcomes of hemorrhagic stroke in patients with high vs. normal homocysteine levels, further inquiries can be directed to the corresponding author.

## Supplementary material

SUPPLEMENTARY FIGURE 1 Sensitivity analysis plot for mortality.

SUPPLEMENTARY FIGURE 2 Sensitivity analysis plot for functional outcome.

SUPPLEMENTARY FIGURE 3 Sensitivity analysis plot for poor neurological outcome.
