# Supplementary figures and images for: The prognostic value of homocysteine levels in hemorrhagic stroke patients: a systematic review and meta-analysis
Source: Front Neurol. 2025 Apr 28;16:1576453. doi: 10.3389/fneur.2025.1576453 (PMC12068063; doi:10.3389/fneur.2025.1576453)

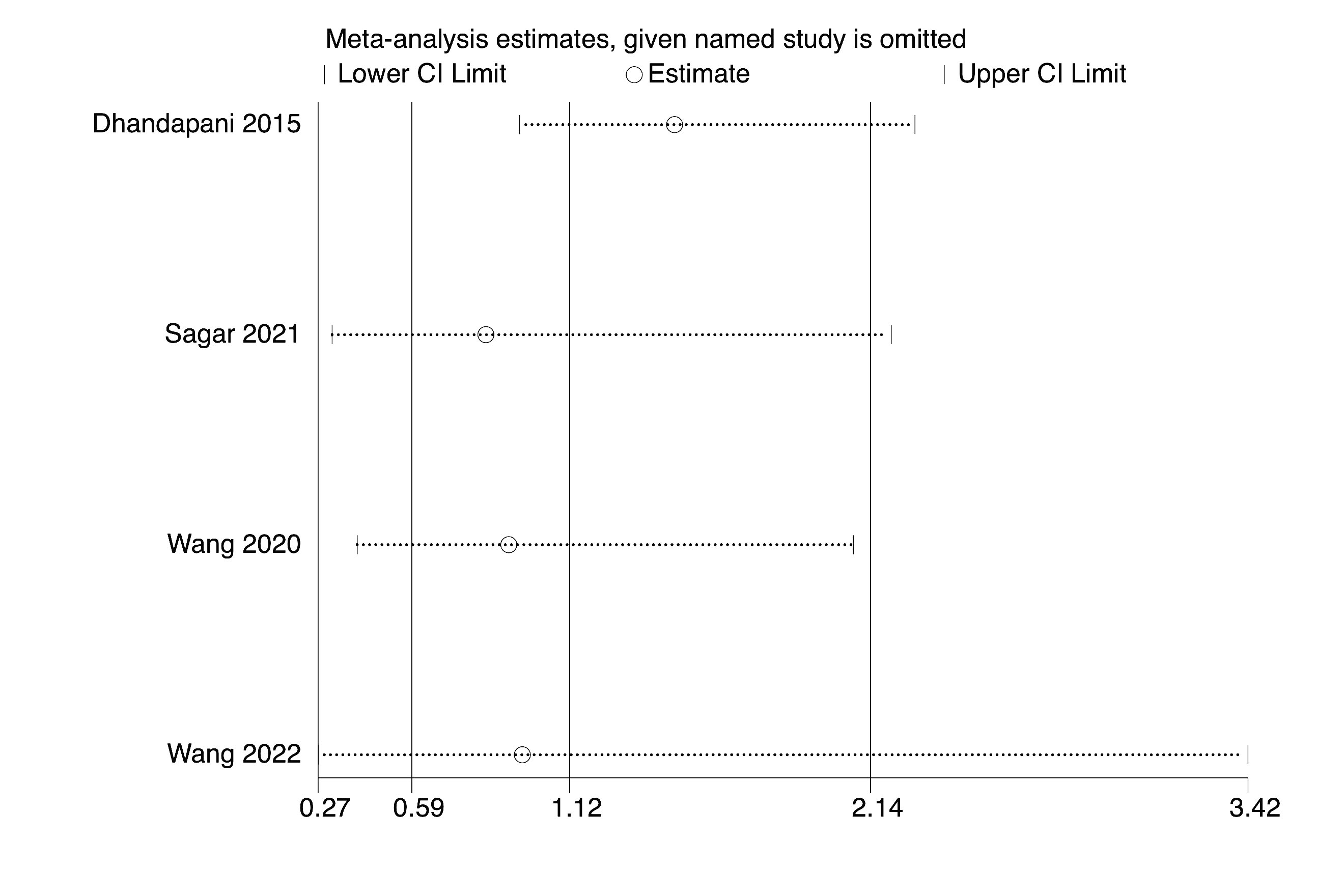

Supplement: SUPPLEMENTARY FIGURE 1 — Sensitivity analysis plot for mortality. [file Image_1.TIF]

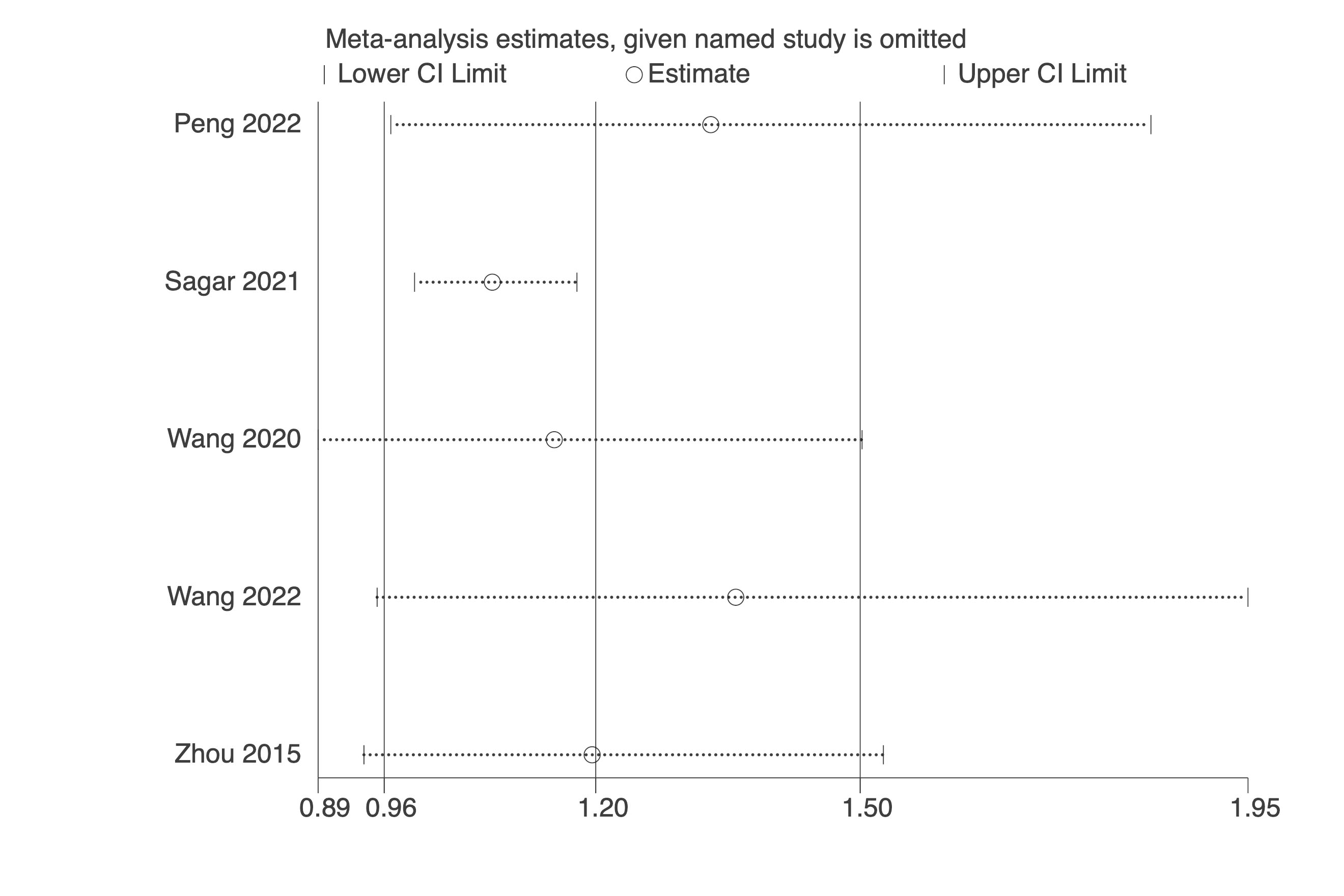

Supplement: SUPPLEMENTARY FIGURE 2 — Sensitivity analysis plot for functional outcome. [file Image_2.TIF]

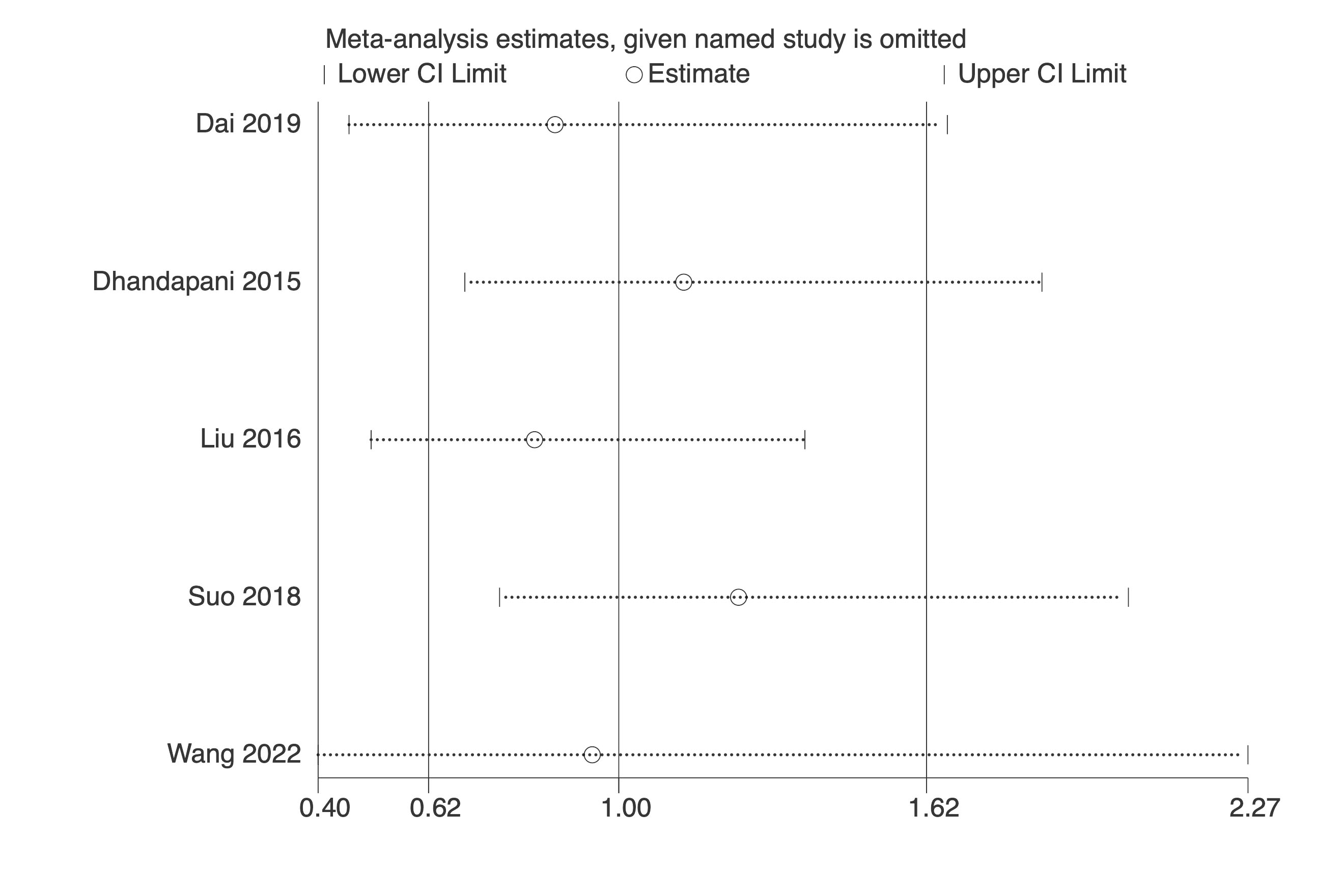

Supplement: SUPPLEMENTARY FIGURE 3 — Sensitivity analysis plot for poor neurological outcome. [file Image_3.TIF]
